# Predicting Cognitive Function from Clinical Measures of Physical Function and Health Status in Older Adults

**DOI:** 10.1371/journal.pone.0119075

**Published:** 2015-03-03

**Authors:** Niousha Bolandzadeh, Konrad Kording, Nicole Salowitz, Jennifer C. Davis, Liang Hsu, Alison Chan, Devika Sharma, Gunnar Blohm, Teresa Liu-Ambrose

**Affiliations:** 1 Experimental Medicine Program, University of British Columbia, Vancouver, British Columbia, Canada; 2 Djavad Mowafaghian Centre for Brain Health, University of British Columbia, Vancouver, British Columbia, Canada; 3 Centre for Hip Health and Mobility, Vancouver Coastal Health Research Institute, Vancouver, British Columbia, Canada; 4 Aging, Mobility, and Cognitive Neuroscience Laboratory, Department of Physical Therapy, University of British Columbia, Vancouver, British Columbia, Canada; 5 Rehabilitation Institute of Chicago, Northwestern University, Chicago, Illinois, United States of America; 6 Department of Biomedical Engineering, Marquette University, Milwaukee, Wisconsin, United States of America; 7 School of Population and Public Health, University of British Columbia, Vancouver, British Columbia, Canada; 8 Centre for Clinical Epidemiology and Evaluation, Vancouver, British Columbia, Canada; 9 Department of Biomedical and Molecular Sciences, Queen’s University, Kingstone, Ontario, Canada; "Mario Negri" Institute for Pharmacological Research, ITALY

## Abstract

**Introduction:**

Current research suggests that the neuropathology of dementia—including brain changes leading to memory impairment and cognitive decline—is evident years before the onset of this disease. Older adults with cognitive decline have reduced functional independence and quality of life, and are at greater risk for developing dementia. Therefore, identifying biomarkers that can be easily assessed within the clinical setting and predict cognitive decline is important. Early recognition of cognitive decline could promote timely implementation of preventive strategies.

**Methods:**

We included 89 community-dwelling adults aged 70 years and older in our study, and collected 32 measures of physical function, health status and cognitive function at baseline. We utilized an L1–L2 regularized regression model (elastic net) to identify which of the 32 baseline measures were strongly predictive of cognitive function after one year. We built three linear regression models: 1) based on baseline cognitive function, 2) based on variables consistently selected in every cross-validation loop, and 3) a full model based on all the 32 variables. Each of these models was carefully tested with nested cross-validation.

**Results:**

Our model with the six variables consistently selected in every cross-validation loop had a mean squared prediction error of 7.47. This number was smaller than that of the full model (115.33) and the model with baseline cognitive function (7.98). Our model explained 47% of the variance in cognitive function after one year.

**Discussion:**

We built a parsimonious model based on a selected set of six physical function and health status measures strongly predictive of cognitive function after one year. In addition to reducing the complexity of the model without changing the model significantly, our model with the top variables improved the mean prediction error and R-squared. These six physical function and health status measures can be easily implemented in a clinical setting.

## Introduction

The world’s population is aging at a rapid rate [1], projecting a significant increase in the number of older adults with cognitive impairment and dementia in the coming decades. Current research suggests that the neuropathology of dementia—including brain changes leading to memory impairment and cognitive decline—is evident years before the onset of this disease [2]. Both cross-sectional and longitudinal evidence have indicated the effect of aging on various domains of cognition after the age of 55 [3]. Older adults with cognitive decline have reduced functional independence and quality of life, and are at greater risk for developing dementia [4]. Therefore, identifying biomarkers that can be easily assessed within the clinical setting and predict cognitive decline is of prime importance [5]. Early recognition of cognitive decline would promote timely implementation of preventive strategies.

Current research efforts have primarily focused on predicting cognitive decline using neuroimaging—including functional and structural magnetic resonance imaging [6,7], positron emission tomography [8,9] and electroencephalographic activity [10]—and cerebrospinal fluid biomarkers [11]. However, these biomarkers are costly and resource intensive, and are thus not widely feasible within clinical settings. Therefore, there is an increasing demand to identify biomarkers that can be widely adopted and used by clinicians.

We propose that potential biomarkers are measures of physical function, in particular mobility [12]. Recent studies have demonstrated significant associations between cognitive function and mobility [13]. Specifically, it is now widely recognized that gait depends on both higher level cognitive processes and sensorimotor processes [14,15]. Higher level cognitive processes are known as executive functions and these processes include the ability to concentrate, to attend selectively, and to plan and to strategize. Reduced executive functions are associated with impaired gait [16–19] and falls [20–24]. Of particular relevance to our study, recent evidence suggests that impaired mobility precedes cognitive decline in older adults [25,26]. Buracchio and colleagues [25] found that decline in gait speed was evident 12 years in older adults before the onset of mild cognitive impairment. They concluded that gait speed may be useful in detecting preclinical dementia.

In this regard, using appropriate statistical methods, we aimed to identify a set of clinical measures of physical and health status that are predictive of cognitive function after one year. We purposefully included measures that: 1) require minimal resources and training to implement; and 2) have established reliability and validity. This inclusion was not based on variables’ correlations with cognitive function.

## Methods

### Study Design and Participants

We included 89 community-dwelling adults aged 70 years and older who completed a 12-month prospective study aimed at investigating the interaction between cognitive function and mobility. Participants were recruited from metropolitan Vancouver via newspaper advertisements. Individuals were eligible if they: 1) were aged 70 to 80 years; 2) scored > 24/30 on the Mini-Mental State Examination (MMSE) [27]; 3) were right hand dominant as measured by the Edinburgh Handedness Inventory [28]; 4) were living independently in their own homes; 5) had visual acuity of at least 20/40, with or without corrective lenses; and 6) provided informed consent. We excluded those who: 1) had a neurodegenerative disease, stroke, dementia (of any type), or psychiatric condition; 2) had clinically significant peripheral neuropathy or severe musculoskeletal or joint disease; 3) were taking psychotropic medication; 4) had a history indicative of carotid sinus sensitivity; or 5) were living in a nursing home, extended care facility, or assisted-care facility.

Ethics approval was obtained from the Vancouver Coastal Research Health Institute and University of British Columbia’s Clinical Research Ethics Board. All participants provided written consent.

### Measurement

We assessed cognitive function at baseline and 12 months. In addition to the cognitive function at baseline, we also collected 31 baseline measures in our model (i.e., a total of 32 baseline measures: four basic descriptors, 18 measures of physical function, nine measures of health status, and one measure of cognitive function). All assessors were trained and standardized protocols were used. For basic descriptors, we measured age in years, standing and sitting height in centimeters, and mass in kilograms.

#### Primary Dependent Variable: Global Cognitive Function

We used the Montreal Cognitive Assessment (MoCA) [29] test to assess global cognitive function. This test assesses multiple domains of cognitive function, including executive functions, attention, language, memory, and orientation, in a short 30-point test. The MoCA has good internal consistency and test-retest reliability and correctly identified 90% of a large sample of individuals with mild cognitive impairment from two different clinics with a cut-off score of < 26/30 [29].

#### Independent Variables (Predictors): Physical Status (Mobility, Balance, Falls Risk, and Fitness)

A total of 18 independent variables were extracted from the following measures of physical status.

The Short Physical Performance Battery (SPPB) [30]: For the Short Physical Performance Battery, participants were assessed on performances of standing balance, walking, and sit-to-stand. Each component is rated out of four points, for a maximum of 12 points; a score < 9/12 predicts subsequent disability [31]. In our analysis, we included six measures derived from the SPPB: 1) walking time over a distance of 4 meters, at usual speed; 2) walking score, based on a participant’s ability (4 points maximum) to walk a distance of 4 meters; 3) sit-to-stand time for a set of five repetitions of rising from a chair and sitting down; 4) sit-to-stand score, based on participant’s ability (4 points maximum) to perform five repetitive chair stands; 5) standing score, based on participant’s standing balance (4 points maximum); and 6) the total SPPS score (12 points maximum), based on all the subcomponents.

The Physiological Profile Assessment (PPA) [32]: Physiological falls risk was assessed using the short form of the PPA. The PPA is a valid and reliable measure of falls risk [32]. Based on a participant’s performance in five physiological domains—postural sway, reaction time, strength, proprioception, and vision—the PPA computes a falls risk score (standardized score) that has a 75% predictive accuracy for falls among older people [33,34]. A PPA Z-score of ≥ 0.60 indicates high physiological falls risk [35].

For our analysis, we used 8 measures derived from the PPA: 1) visual contrast sensitivity, using The Melbourne Edge Tests (MET). Participants were presented with 20 circular patches containing edges with reducing contrast and variable orientation. The circle with lowest contrast—in which participants can correctly identify the orientation of the edge—is considered their MET score. This test has high test-retest reliability [36] and good external validity as a predictor of falls [37]; 2) average proprioception score. We asked seated participants (closed eyes) to align their feet on either side of a thick clear sheet. The difference in matching the great toes (in degrees) is considered as proprioception score. We calculated the average from five trials; 3) average hand reaction time. We used a light as the stimulus and asked participants to click on a mouse for 10 times. The average of the 10 trials was considered as the reaction time; 4) best dominant quadriceps strength. We measured the strength of quadriceps while the participants were seated. The best score was selected among three trials; 5) average quadriceps strength from three trials; 6) postural sway on foam. We used a swaymeter that measures displacements of the body at waist level. The device consisted of a 40cm long rod (attached to participants) with a vertically mounted pen at its end. The participants were asked to stand still on an high-density foam with open eyes, and the pen recorded their sway; 7) postural sway on the floor. We repeated the swaymeter measurements on the floor; and 8) overall falls risk score, by combining all the subcomponents of PPA assessment.

The Activities-specific Balance Confidence (ABC) [38]: Based on the self-efficacy theory by Tinetti and colleagues [39], The ABC questionnaire was used to measure an aspect of the psychological impact of balance impairment and/or falls. The participants were asked to rate their confidence in performing each of the activities on a scale from 0 (no confidence) to 100% (complete confidence) without losing balance or becoming unsteady. We used the mean ABC score, calculated by averaging all the percentages for each of the 16 items.

Timed Up and Go (TUG) [40]: This test was used to assess functional mobility. Each participant was timed while he rose from a chair, walked 3 meters, turned, walked back, and sat down again. We repeated this test for two times and used the average.

The Physical Activity Scale for the Elderly (PASE) [41]: This test is comprised of self-reported movement counts for occupational, household and leisure items over a one-week period. We used the total PASE score.

Six-Minute Walk Test: We assessed physical fitness by the Six-Minute Walk Test (6-MWT) [42]—a walking test of general cardiovascular capacity in older adults [43]. The total distance walked in meters in six minutes was recorded.

#### Independent Variables (Predictors): Health Status

A total of nine independent variables were extracted from the following measures of health.

Cardiovascular risk: We measured hip girth and waist girth in centimeters and then calculated the waist-to-hip ratio using the formula (waist girth / hip girth). The waist-to-hip ratio is a measure of obesity and cardiovascular risk [44]. Resting heart rate, heart rate immediately post 6MWT, and resting blood pressure were recorded in duplicate, using a voscillometric sphygmomanometer, the Omron HEM-775 Values were presented as an average of two recordings that were taken one minute apart.

Mood: We used the Geriatric Depression Scale (GDS), a 30-point self-rating test, to assess depression in our population. The GDS is a reliable and validated basic screening measure for depression in older adults [45].

Comorbidities: Comorbidities were assessed with the Functional Comorbidity Index (FCI) [46], a 21-item questionnaire that calculates the total number of comorbidities [46].

### Statistical Method

#### Variable Selection

We used a regularized regression model (elastic net) from the Matlab Statistics Toolbox (2012b, The Mathworks, Inc., Natick, Massachusetts, United States) to explore the relation between the 32 baseline measures and cognitive function after one year. Elastic net is an automated shrinkage and penalized statistical method that reduces variability in the estimates of regression coefficients. In elastic net, regularization parameters of L1 (i.e., positive weighting parameter that promotes shrinkage in the regularized regression coefficients) and L2 (i.e., weighting parameter that promotes stability on regularization and protects the fitting from collinearity) are introduced into the standard multiple linear regression model to shrink some coefficients to exactly zero. For a given lambda (i.e., the L1 weighting parameter) and an alpha between 0 and 1 (i.e., the L2 weighting parameter), elastic net minimizes the error, as shown below.

$$\text{Min}\frac{1}{2N}\;[{\left||\text{Y}-\beta_{0}-\text{X}\;\beta |\right|}^{2}\;+\;\lambda ||\beta_{1}||\;+\;\frac{\left(1-\alpha \right)}{2}\;\lambda {\left||\beta_{2}|\right|}^{2}],$$

where

$$||\beta_{1}||=\sum_{j=1}^{p}\;\beta_{j}\;\text{and}\;{\left||\beta_{2}|\right|}^{2}=\sum_{j=1}^{p}\;{\beta^{2}}_{j}$$

Here, Y represents cognitive function after one year for our 89 participants, X is a 89*32 (participants * independent variables) matrix of physical function and health status measures, N is the number of participants, and p is the predictors. To be able to compare the coefficients and meet elastic net’s assumptions, the independent variables are standardized (i.e., converted to Z-Scores), and the dependent variable is mean-centred. To evaluate the quality of the model, we used standard jack-knife (leave one out cross-validation procedures). Elastic net regression is a preferred alternative to conventional variable selection methods, such as stepwise regression, that have been criticized for their bias, over-fitting, and exaggerated p-values [47].

**Fig 1:**
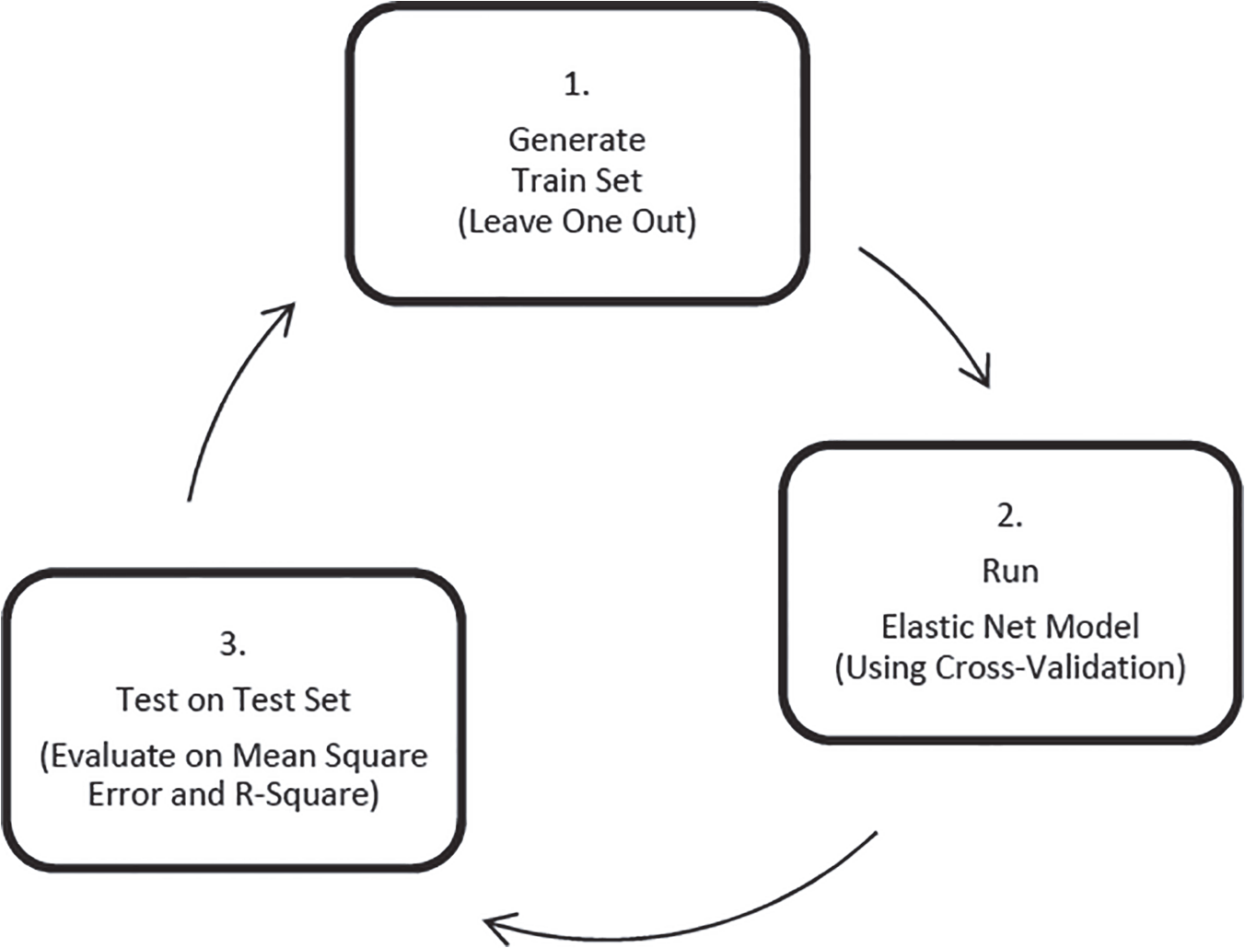
The process of variable selection using the elastic net method. Step 1 shows the generation of train and test sets for each cross-validation loops. We used jackknifing technique to assign one participant to the test set and the rest to the training set. In Step 2, the optimized model is estimated for the training set, using elastic net method. This model has minimized squared error on each cross-validation loop. This model is then tested on the test set in Step 3. After the whole process is repeated over all the participants to avoid the bias, we selected the variables which were consistently selected on all the cross-validation loops. There were 6 variables which were selected in this way.

The process of variable selection using the elastic net method is illustrated in **Fig. 1**. With Jack-knife resampling technique (Steps 1 to 3; **Fig. 1**), the complete process was repeated for each participant in the X matrix separately (i.e., 89 times), to reduce the bias in selecting the minimized error. On each run, one participant was assigned to the testing set, and the rest was assigned to the training set (Step 1; **Fig. 1**). Then a Leave-One-Out Cross-Validation (LOOCV) was performed within the training set to select the tuning parameters, which minimize the Mean Squared Error on the training set (Step 2; **Fig. 1**).

At the end of the variable selection process, we had 89 sets of selected variables with their corresponding coefficients. Six variables were consistently selected in all the 89 sets. We built our final model on top of these six variables, since they had all contributed to minimizing mean squared error in each cross-validation loop.

#### Model Construction and Testing

At the end of each cross-validation loop, we built three linear regression models: 1) based on only one variable of baseline MoCA, 2) based on the six variables selected in every cross-validation loop, and 3) a full model based on all the 32 variables. This step was added to enable us to compare the models by their R-squares.

## Results

The mean (SD) age of our population was 76 (3.12) years. The demographics and characteristics of the 89 participants are presented in Table 1. 58% of our sample population were women. Based on the mean baseline MoCA score (24.16), our participants had mild cognitive impairment. On average, performance on the MoCA reduced by 0.40 over the 12-month study period. Based on the mean baseline PPA score (0.37), our participants were not at high risk for falls.

**Table 1:** Descriptive statistics for the outcome measure and 32 predictors.

	N	Minimum	Maximum	Mean	Std. Deviation	Mean Coefficients
Age (year)*	89	70	82	76.07	3.12	−0.29
Weight (kg)	89	45.20	130.15	74.07	16.10	0
Height (cm)	89	147.60	186.45	165.19	8.63	0
Sitting Height (cm)	89	121.13	688.60	136.59	59.33	0.08
Hip Girth (cm) *	89	66.93	148.63	94.15	14.15	0.36
Waist Girth (cm)	89	85.70	142.43	105.94	10.69	0.10
Waist to Hip Ratio	89	.70	1.22	.88	.08	0
Total PPA Score	89	−1.29	2.81	.37	.90	−0.18
Visual Contrast Score (dB)	89	12	24	20.08	2.09	0
Mean Proprioception	89	.0	6.40	1.60	1.24	0
Mean Reaction Time (ms)	89	163.9	369.10	232.44	38.05	0
Mean Quadriceps Strength (kg) *	89	4.33	46	27.43	9.17	−0.29
Best Quadriceps Strength (kg)	89	5	46	29.05	9.37	−0.10
Floor Sway (mm) *	89	33.67	180.98	65.35	30.26	−0.13
Foam Sway (mm)	89	55.14	557.41	145.61	92.54	−0.05
Mean ABC Score	89	38.12	100.00	85.54	13.20	0.08
GDS	89	0	6	.53	1.24	−0.13
FCI	89	0	8	3.01	1.89	0
Baseline MoCA*	89	15	30	24.16	3.21	1.61
Mean TUG (s)	89	4.85	23.88	8.09	2.48	−0.06
SPPB Standing Score	89	2	4	3.67	.61	0
SPPB Walking Score	89	2	4	3.94	.27	0
SPPB Walking Time (s)	89	2.08	6.50	3.37	.69	0
SPPB Sit to Stand Score	89	1	4	2.99	1.12	0
SPPS Sit to Stand Time (s)	89	6.21	42.95	12.66	5.08	0
SPPB Total Score	89	5	12	10.61	1.57	0
Rest Heart Rate	89	45	103	72.21	13.03	0.02
Post Exercise Heart Rate	89	52	120	85.09	16.26	−0.17
Resting Blood Pressure—Systolic (mm Hg)	89	13	205	145.33	25.19	0
Resting Blood Pressure—Diastolic (mm Hg)	89	61	106	80.04	10.10	0
Six Minute Walk Test (m)	89	180	690	488.46	95.61	0
PASE*	89	.00	489.89	123.88	64.33	0.30
Outcome: Final MoCA	89	12	30	23.75	3.49	N/A

Abbreviations: PPA: Physiological Profile Assessment, GDS: Geriatric Depression Score, FCI: Functional Comorbidity Index, MoCA: Montreal Cognitive Assessment, TUG: Timed Up & Go, SPPB: Short Physical Performance Battery, PASE: Physical Activity Scale for Elderly.

* presents The six variables consistently selected in all models.

Mean coefficients are calculated based on coefficients obtained from elastic net.

### Statistical Analysis

First, we wanted to ask which variables would be most useful for our predictions. Using elastic net, the six baseline variables consistently selected on every cross-validation loop were reported as best predictors of cognitive decline, as measured by MoCA, over the 12-month study period. The following list is sorted in the order of their contribution to the variance of MoCA: 1) baseline MoCA, 2) hip girth, 3) PASE, 4) age, 5) mean quadriceps strength, and 6) postural sway on the floor.

### Model Evaluation

To evaluate our model, we constructed three linear regression models in each cross-validated loop; one using only one variable of baseline MoCA (Model 1), the second one using the six variables consistently selected across all the cross-validation loops (i.e., Model 2), and the third one using all the 32 variables (i.e., Model 3). Model 2 had the smallest mean squared errors (Table 2; mean = 7.47) than Model 1 or 3 (Table 2; mean = 7.98 and 115.33). Moreover to the improved mean squared errors, model 2 explained 47% of the variance on MoCA after one year, which was significantly higher than that of model 1 (i.e., 37%) by 10% (p<0.001).

**Table 2:** The mean squared errors associated with Model 1, Model 2, and Model 3, to compare the model with top 10 variables selected at each cross-validation loop with the model with only one variable of baseline MoCA and also with model with all the 32 variables.

Models	Mean Squared Error	SD	Min	Max	R-Squared
Model 1	7.98	11.98	0.0002	76.50	37%
Model 2	7.47	12.84	0.001	83.43	47%
Model 3	115.33	960.19	0.001	9070.21	59%

Model 1: Standard Regression Model with One Variable of Baseline MoCA.

Model 2: Standard Regression Model with Six Variables Consistently Selected at Every Cross-Validation Loop.

Model 3: Standard Regression Model Using All 32 Variables.

Abbreviations: MoCA: Montreal Cognitive Assessment.

## Discussion

Worldwide, one new case of dementia is detected every four seconds [48]. Thus, there is a concentrated effort to identify patient characteristics, or biomarkers, that are predictive of conversion. Early recognition of cognitive decline would promote timely implementation of preventive strategies. Ideally, such biomarkers could be assessed in a wide variety of clinical settings with minimal resources.

Using a statistical approach, we identified a set of six clinical measures that predicted cognitive function after one year among community-dwelling older adults. The six clinical measures were broadly of: 1) falls risk, 2) muscular strength, 3) cardiovascular function, and 4) physical activity. Overall, our results concur with previous studies that examined factors associated with healthy aging [49,50] and more specifically, cognitive health in older adults.

The prevalence of impaired mobility is 35% for community-dwelling older adults of age 70 years and older [51]. Falls are a significant consequence of impaired mobility. One of the key factors contributing to falls is impaired cognitive function. Even mild cognitive decline in otherwise healthy community-dwelling older adults is a significant risk factor for falls [24]. It is critical to recognize that the relationship between mobility and cognitive function is not unidirectional (i.e., impaired cognitive function leads to falls), but rather it is bidirectional [52,53]. Thus, there is growing recognition that clinical gait abnormalities and falls are early biomarkers of cognitive impairment and dementia. For example, gait speed was reported to slow a decade before the diagnosis of MCI [25]. Thus, our finding of falls risk (i.e., postural sway) as a predictor of cognitive function concur with current evidence.

The measure of dominant quadriceps strength (i.e., average strength) was among the six clinical measures that predicted cognitive function. This concurs and extends the prospective cohort study of Boyle and colleagues [54], who demonstrated that in an average follow-up time of 3.6 years, greater muscle strength was significantly associated with a slower rate of global cognitive decline in a cohort of 900 community-based older adults.

Both hip girth and physical activity were consistently selected in all the elastic net models over the jack-knife process. Heitmann and colleagues [55] recently demonstrated that a large hip circumference has an independent and beneficial positive effect on both cardiovascular health and mortality in middle-aged adults. Cardiovascular function is highly associated with cognitive function and dementia risk [56]. Reduced cardiovascular morbidity should be associated with better cognitive 0070erformance [57].

Epidemiological studies demonstrate a consistent relationship between higher physical activity levels and a reduced risk of developing dementia [58–61]. A meta-analysis of 16 prospective, epidemiological studies on the incidence of neurodegenerative disease, found more physical activity at baseline reduced the risk of developing all-cause dementia by 28% and of developing AD by 45%, even after controlling for confounding variables [62]. Thus, it is not surprising physical activity was a significant predictor of cognitive function in our study.

We recognize the limitations of this study. Our model with the six variables is marginally performing better than the model with baseline cognitive function. We speculate that for predictions of longer than one year, additional features might be selected, resulting in lower prediction errors. Moreover, in spite of marginal improvement in error and small change of MoCA from baseline to one year, the R-squared was improved by 10%, which might be representative of the greater variance (total of 47%) explained by our model with six selected variables. Lastly, larger datasets and experiments of longer than one year would be needed to investigate the selection of variables separately for men and women, and with higher validity and predictability.

In summary, we built a parsimonious model based on a selected set of six physical function and health status measures strongly predictive of cognitive function after one year. Complex models with a large number of variables have increased risk of over-fitting in predictive analysis. Over-fitted models generally have poor predictive performance, as they can inflate minor fluctuations in the data. Constructing a model with a smaller set of variables will decrease the complexity of the model, will show a better performance when new data is introduced to the model for prediction, and will be more robust to outliers [63]. In addition to reducing the complexity of the model without changing the model significantly, our model with the selected variables improved the mean prediction error (7.47 vs. 115.33 with all the 32 variables) and the R-squared (47% vs. 37% with only baseline MoCA). This result further proves the robustness of our model using fewer variables. These six physical function and health status measures can be easily implemented in a clinical setting.
